# Beyond Testis Size: Links between Spermatogenesis and Sperm Traits in a Seasonal Breeding Mammal

**DOI:** 10.1371/journal.pone.0139240

**Published:** 2015-10-02

**Authors:** Eliana Pintus, José Luis Ros-Santaella, José Julián Garde

**Affiliations:** 1 Department of Veterinary Clinics and Pathology, Faculty of Veterinary Medicine, University of Sassari, Sassari, Italy; 2 Department of Veterinary Sciences, Faculty of Agrobiology, Food and Natural Resources, Czech University of Life Sciences, Prague, Czech Republic; 3 Department of Animal Science and Food Processing, Faculty of Tropical AgriSciences, Czech University of Life Sciences, Prague, Czech Republic; 4 SaBio IREC (CSIC–UCLM–JCCM), Campus Universitario, Albacete, Spain; Zhejiang University, CHINA

## Abstract

Spermatogenesis is a costly process that is expected to be under selection to maximise sperm quantity and quality. Testis size is often regarded as a proxy measure of sperm investment, implicitly overlooking the quantitative assessment of spermatogenesis. An enhanced understanding of testicular function, beyond testis size, may reveal further sexual traits involved in sperm quantity and quality. Here, we first estimated the inter-male variation in testicular function and sperm traits in red deer across the breeding and non-breeding seasons. Then, we analysed the relationships between the testis mass, eight parameters of spermatogenic function, and seven parameters of sperm quality. Our findings revealed that the Sertoli cell number and function parameters vary greatly between red deer males, and that spermatogenic activity co-varies with testis mass and sperm quality across the breeding and non-breeding seasons. For the first time in a seasonal breeder, we found that not only is the Sertoli cell number important in determining testis mass (r = 0.619, *p* = 0.007 and r = 0.248, *p* = 0.047 for the Sertoli cell number assessed by histology and cytology, respectively), but also sperm function (r = 0.703, *p* = 0.002 and r = 0.328, *p* = 0.012 for the Sertoli cell number assessed by histology and cytology, respectively). Testicular histology also revealed that a high Sertoli cell number per tubular cross-section is associated with high sperm production (r = 0.600, *p* = 0.009). Sperm production and function were also positively correlated (r = 0.384, *p* = 0.004), suggesting that these traits co-vary to maximise sperm fertilisation ability in red deer. In conclusion, our findings contribute to the understanding of the dynamics of spermatogenesis, and reveal new insights into the role of testicular function and the Sertoli cell number on testis size and sperm quality in red deer.

## Introduction

Spermatogenesis is a complex developmental process by which diploid spermatogonia generate haploid spermatozoa through a series of cyclic and highly coordinated events known as spermatocytogenesis, meiosis, spermiogenesis, and spermiation [1]. Each step is fundamental since defects that occur in any one of them can result in the failure of the entire process, leading to the production of defective spermatozoa and a reduction or absence of sperm production [2]. In amniotes (i.e. reptiles, birds, and mammals), spermatogenesis occurs in seminiferous tubules which possess a permanent population of Sertoli cells and spermatogonia (reviewed by [3]). Whereas spermatogonia act as a reservoir for the succeeding bouts of spermatogenic activity [3], Sertoli cells have the role of supporting, nursing, and regulating germ-cell function, permitting them to migrate from basal to apical positions as spermatogenesis proceeds [4]. Sertoli cells play a central role in the development of a functional testis because without their physical and metabolic support, germ-cell differentiation, meiosis, and transformation into spermatozoa would not occur [5, 6]. In the adult male, Sertoli cells determine testis size and daily sperm production [6, 7], but they may also account for the intra-male variation in sperm size [8]. Recently, Rajak *et al*. [9] found that, in adult bulls, the Sertoli cell index was positively associated with ejaculate sperm concentration, mass activity, individual motility, viability, and membrane integrity. Nevertheless, the relationship between the Sertoli cell traits and sperm quality has so far received no empirical testing in seasonal breeders, although these are valuable models for the study of seasonal regulation of testis function [10].

Even though the links between the testicular morphology and sperm traits have been explored in comparative studies [11–14], little is known at the intra-specific level [9, 15], in spite of the major functional significance. Studies using intra-specific variation in testes investment are powerful and instructive also from an evolutionary perspective because they may reveal the underlying processes governing any observed inter-specific pattern [16]. Because both sperm quantity and quality are regulated by spermatogenesis, an understanding of this process provides useful insight into adaptations of, and constraints on, testicular function and sperm production, in relation to sperm competition [13]. What indeed makes a spermatozoon successful at fertilisation depends to a large extent on the features of the environment in which spermatozoa compete [17, 18], and also on the efficiency of the machinery by which it was produced.

Across the Cervidae family, red deer show considerable testes investment relative to their body size [19] and elaborate secondary sexual traits, which in turn are related to sperm production and quality [20]. Additionally, the short period of female sexual receptivity (usually no more than 24 h) and to some extent female promiscuity [21], are likely to impose an intense competition on males, which results in a large degree of male fertility [22]. The investment in spermatogenesis is therefore expected to be high and diverse among red deer males.

In red deer from the southern Iberian Peninsula, the breeding season lasts from September to December [20] (and references therein). During this phase, red deer show the highest values for Sertoli cell number and spermatogenic competence [23], together with the greatest testis size and sperm quality [24]. Due to the fact that the Sertoli cell number decreases across the breeding and non-breeding seasons [23], we hypothesised that changes in the Sertoli cell number directly translate into variation in testis mass and sperm quality. To test our hypothesis, we collected data from natural populations of Iberian red deer across the breeding and non-breeding seasons. The annual cycle of testicular involution and recrudescence makes deer valuable models in which to study the basic mechanisms for the regulation of gonadal function [10, 15]. On the other hand, a comprehensive approach that simultaneously considers the machinery, products, and scheduling of spermatogenesis will be crucial to more fully understand the evolution of the most fundamental of male reproductive traits [25]. Therefore, the aims of this study were i) to quantify the inter-male variation of testicular and sperm traits, and ii) to assess the links between the Sertoli cell traits, spermatogenic competence, testis size, and sperm parameters across different phases of the reproductive cycle. Spermatogenesis is a costly process, and we predicted that the Sertoli cell number and spermatogenic competence maximise sperm quantity and quality during the breeding season, when sperm demand and the chances for mating success are expected to be maximal.

## Materials and Methods

This study was approved by the “Comité de Ética en Investigación de la Universidad de Castilla-La Mancha”. All animals were handled in accordance with Spanish Animal Protection Regulation RD53/2013, which conforms to the European Union Regulation 2003/65/CE. Stags were legally hunted in their natural habitat in accordance with the harvest plan of the game reserve. The harvest plans were made in accordance with Spanish Harvest Regulation, Law 2/93 of Castilla-La Mancha, which conforms to the European Union regulations. Landowners and managers of the red deer populations gave permission to the authors to use the samples.

### Chemicals and solutions

All of the reagents were purchased from Sigma-Aldrich (Madrid, Spain), unless otherwise indicated.

A Salomon’s modified extender Fraction A contained: Tris (2.70%, w/v), fructose (1%, w/v), citric acid (1.4%, w/v), and clarified egg yolk (20%, v/v) (pH 6.8, osmolality 300 mOsm/Kg) [26]. Bovine gamete medium (BGM-3) was composed of 87 mmol/L NaCl, 3.1 mmol/L KCl, 2 mmol/L CaCl_2_, 0.4 mmol/L MgCl_2_, 0.3 mmol/L NaH_2_PO_4_, 40 mmol/L HEPES, 21.6 mmol/L sodium lactate, 1 mmol/L sodium pyruvate, 50 μg/mL kanamicine, 10 μg/mL phenol red, and 6 mg/mL BSA (Bovine Serum Albumine) (pH 7.5), as previously described [27].

### Data collection

Samples were collected from 47 red deer *(Cervus elaphus)* stags (age >4.5 years) culled from November 2010 to February 2011 in southern Spain. Samples could not be collected during the remainder of the year because those times were beyond the hunting season for this species. Testes, within the scrotal sac, were cut with a knife and transported to the laboratory in a plastic bag at ambient temperature. Samples were processed between 4 and 8 h after the death of the animals, a time lapse during which epididymal sperm characteristics do not change significantly [28]. In order to examine the dynamics of spermatogenesis and sperm traits throughout the breeding and non-breeding seasons, the samples were divided into three groups, as previously reported [23]: the breeding season group (BS; from late November to late December, *n* = 17), the post-breeding season group (PB; from early January to early February, *n* = 16), and the non-breeding season group (NB; late February, *n* = 14). Part of the dataset used in this study concerning testis mass and testicular parameters was exploited and published in our previous study [23] to examine the agreement between testicular histology and fine needle aspiration cytology (FNAC) and the seasonal variation of the spermatogenic and the Sertoli cell number in red deer.

### Assessment of testis mass and spermatogenic competence

Testis mass was recorded to the nearest 0.01 g using an electronic balance (EK-400H balance; A&D Co., Tokyo, Japan) after removing the epididymis and the spermatic cord with a surgical blade. Testis mass was not controlled for by allometry because male body condition declines during the rutting season [19]. Moreover, in species with alternative reproductive tactics, such as the red deer (e.g. harem-defence or sneaking tactics; [19]), relative testis mass varies according to the mating tactic employed (reviewed by [29]).

**Fig 1:**
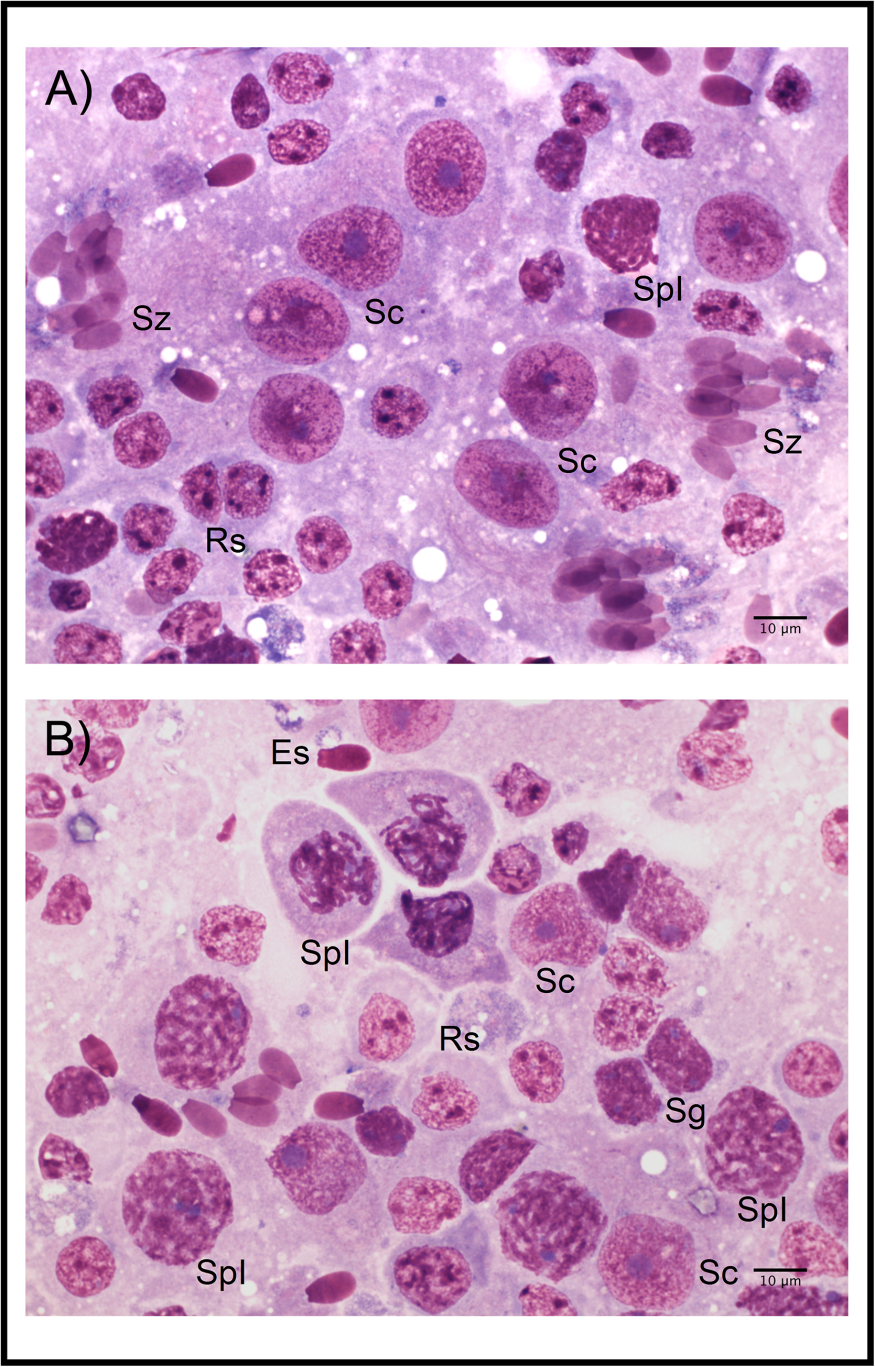
Testicular germ cells and Sertoli cells from red deer. A-B) Sc: Sertoli cells; Sg: spermatogonia; SpI: primary spermatocytes; Rs: round spermatids; Es: elongated spermatids; Sz: spermatozoa.

**Fig 2:**
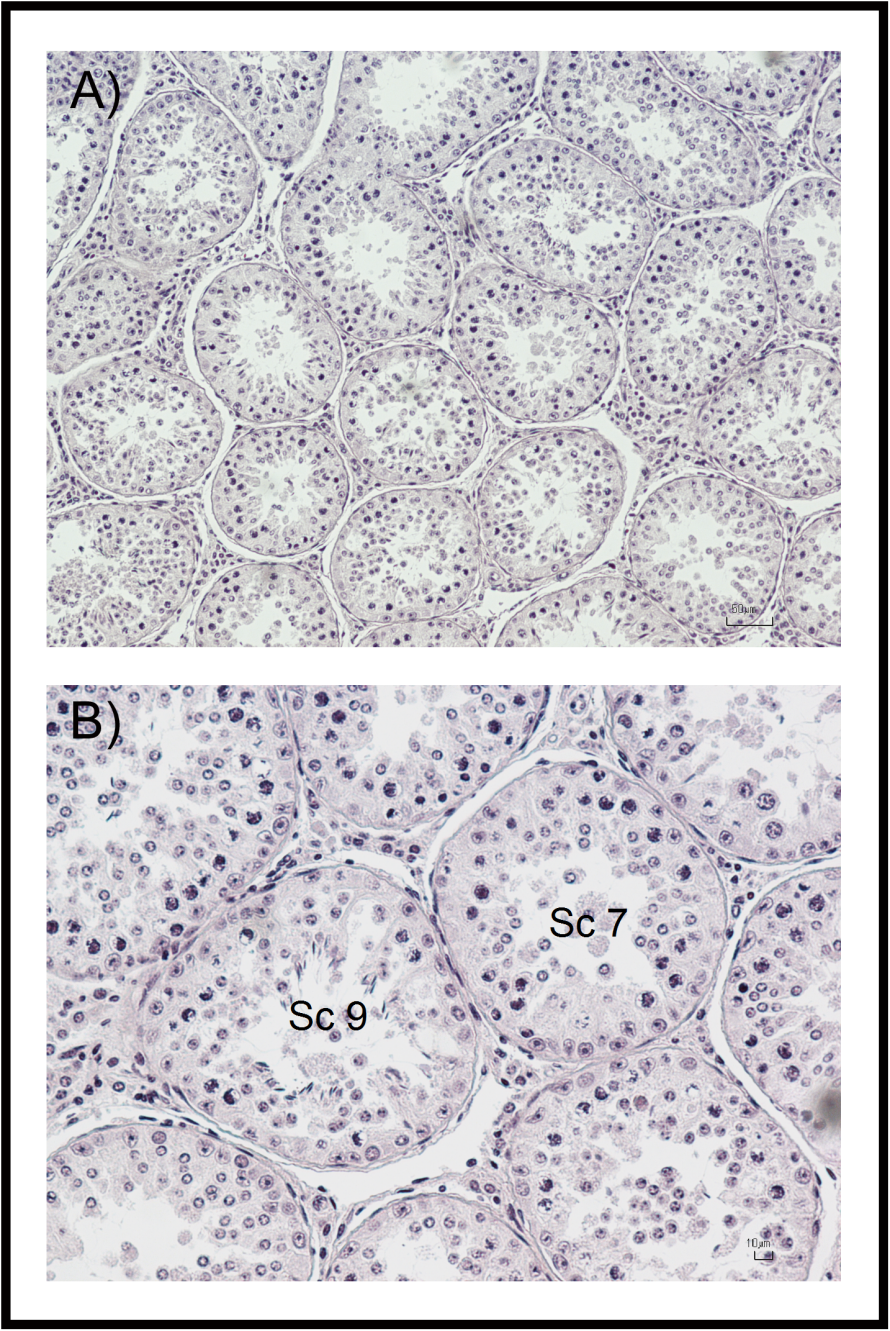
Testicular histology and Johnsen score of red deer. A-B) Sc: Johnsen score.

Testicular histology and cytology were performed as described previously [23]. Briefly, cytological samples were collected using the FNAC technique and stained with Hemacolor® (Merck, Darmstadt, Germany). The smears were examined under oil immersion at 1000X magnification using light microscopy (Nikon Eclipse 80i; Nikon, Kanagawa, Japan). In order to perform a quantitative assessment of spermatogenesis, at least 200 spermatogenic and Sertoli cells were evaluated per testis (Fig 1). Then, six cytological indices were determined as follows: i) the Sertoli cell index (SEI), which is the percentage of Sertoli cells per 100 spermatogenic cells, and estimates the Sertoli cell number ii) the spermatic index (SI), which is the percentage of spermatozoa per 100 spermatogenic cells, and estimates sperm production; iii) the meiotic index (MI), which is the ratio of round spermatids to primary spermatocytes, and estimates the meiotic germ loss; iv) the ratio of elongated spermatids to round spermatids (ES/RS), which estimates the post-meiotic germ loss; v) the ratio of elongated spermatids to total germ cells (ES/GC), which estimates the overall germ loss and the efficiency of spermatogenesis; and vi) the ratio of round spermatids to Sertoli cells (RS/SC), which estimates the Sertoli cell efficiency [13, 23, 30, 31]. In addition, one fragment of testicular tissue (~1 cm^3^) was fixed in modified Davidson’s fixative solution for 24–48 h, then stored in an ethanol:water solution (70:30 v/v) until analysis [32, 33]. The samples were then embedded in paraffin, cut into 4 μm sections and stained with haematoxylin and eosin (H&E). Two histological indices were evaluated: Johnsen score (*N* = 26: in the BS group *n* = 11, in the PB group *n* = 7, and in the NB group *n* = 8, respectively) and the Sertoli cell number per tubular cross-section (SC/TCS; *N* = 15: in the BS group *n* = 5, in the PB group *n* = 3, and in the NB group *n* = 7, respectively), which estimate the spermatogenic activity and the Sertoli cell number, respectively [23, 34]. The Johnsen score and the Sertoli cell number per tubular cross-section were evaluated at 500X magnification using light microscopy on 50 and 10 roundish randomly selected tubular cross-sections per testis, respectively. The Johnsen score was assessed by giving to each tubular section a score from 10 to 1, according to the presence or absence of the main cell types arranged in the order of maturity using the method described by Johnsen [34], as shown by Pintus *et al*. [23] (Fig 2). A smaller subset of the samples was available for histological evaluations, because the time lag between the death of an animal and the fixation of the testes negatively affects the quality of some samples. Therefore, unsuitable samples were excluded from the analysis.

Because no differences were found between the right and left testes in any of the testicular parameters evaluated (S1 Table), testis mass and the spermatogenic parameters were calculated by averaging the values from the left and right testes for each individual. All histological and cytological analyses were performed by a single trained examiner (EP).

### Sperm analysis

Spermatozoa were collected from the epididymal cauda by repeated longitudinal and transverse cuts and placed in 0.5 mL Salamon’s modified extender Fraction A [26]. Sperm from both epididymides were pooled because previous studies demonstrated no differences between them [35]. After the assessment of sperm concentration using a Bürker chamber, the samples were diluted down to 25–30*10^6^ spz/mL and incubated at 37°C for 10 min in order to evaluate sperm motility both subjectively and by a CASA (Computer Assisted Sperm Analysis) system. A pre-warmed Makler (Sefi Medical Instruments, Haifa, Israel) chamber was loaded with 5 μL of sample. For each sample, we subjectively assessed the percentage of individual motile sperm and the quality of motility using a scale from 0 (lowest: immotile or death) to 5 (highest: progressive and vigorous movements). The sperm motility index (SMI) was calculated according to the formula reported previously [36]. Sperm kinetics was assessed by the CASA system composed of an optical phase contrast microscope (Nikon Eclipse 80i, Nikon, Kanagawa, Japan), equipped with negative phase contrast objectives and a warming stage at 37°C, a Basler A302fs camera (Basler Vision Technologies, Ahrensburg, Germany), and a PC with the Sperm Class Analyzer software (SCA2002: Microptic, Barcelona, Spain). The software standard settings were adjusted for deer spermatozoa as follows: 25 frames/s; 20 to 90 μm^2^ for the head area; and VCL >10 μm/s in order to classify a spermatozoon as motile. A total of three kinetic parameters were recorded: average-path velocity (VAP, μm/s), curvilinear velocity (VCL, μm/s), and straight-line velocity (VSL, μm/s). A minimum of 250 sperm per sample were recorded and analysed.

Sperm viability and mitochondrial activity were evaluated as described previously [27]. Briefly, the samples were diluted to a concentration of 10^6^ spermatozoa/mL in BGM-3 solution and stained using three fluorophores. Sperm viability was assessed with 0.1 μmol/L YO-PRO-1 (Invitrogen, Barcelona, Spain) and 10 μmol/L propidium iodide (PI), whereas mitochondrial activity was assessed with 0.1 μmol/L Mitotracker Deep Red. After 20 min of incubation in the dark, the samples were run through a flow cytometer Cytomics FC500 (Beckman Coulter, Brea, CA, USA) furnished with a 488-nm Ar-Ion laser (excitation for YO-PRO-1 and PI), and a 633-nm He-Ne laser (excitation for Mitotracker Deep Red). The FSC (forward-scattered light) and SSC (side-scattered light) signals were used to gate out debris (non-sperm events). Fluorescence from YO-PRO-1 was read using a 525/25BP filter, PI was read using a 615DSP filter, and Mitotracker Deep Red was read using a 675/40BP filter. Fluorescence captures were controlled using the RXP software provided with the cytometer. All of the parameters were read using logarithmic amplification. For each sample, 5000 spermatozoa were recorded, saving the data in flow cytometry standard (FCS) v. 2 files. The analysis of the flow cytometry data was carried out using WEASEL v. 2.6 (WEHI, Melbourne, Victoria, Australia).

The morphology of 200 spermatozoa per animal was assessed to determine the percentage of normal sperm. Spermatozoa were stained with Panoptic (Panreac Quimica SA, Barcelona, Spain) and evaluated at 400X magnification using light microscopy. Sperm morphology was evaluated by a single trained examiner (JLRS).

### Statistical analysis

All statistical analyses were performed using SPSS 20.0 (SPSS Inc, Chicago, IL, USA). The Kolmogorov-Smirnov and Levene’s tests were used to check data normality and homogeneity of variance, respectively. A paired sample T-Test was used when the data were normally distributed, otherwise a Wilcoxon Signed Ranks test was used. One-way ANOVA was used to compare groups using a Tukey post-hoc test in the case of homogeneity of variance, otherwise a Games-Howell post-hoc test was used. A principal component analysis (PCA) was performed in order to reduce many variables to a smaller number of new derived variables which adequately summarized the original information [37]. Bartlett sphericity test and Keiser-Meyer-Olkin (KMO) were assessed as measures of sampling adequacy [38]. Components were rotated (varimax) using the Kaiser normalisation. One-tailed Pearson correlation test and regression analysis were used to assess the relationship between testis mass, spermatogenic competence, and sperm parameters. Each individual record represents an independent sample unit.

## Results

### Testis mass, spermatogenic competence, and sperm parameters in red deer

**Fig 3:**
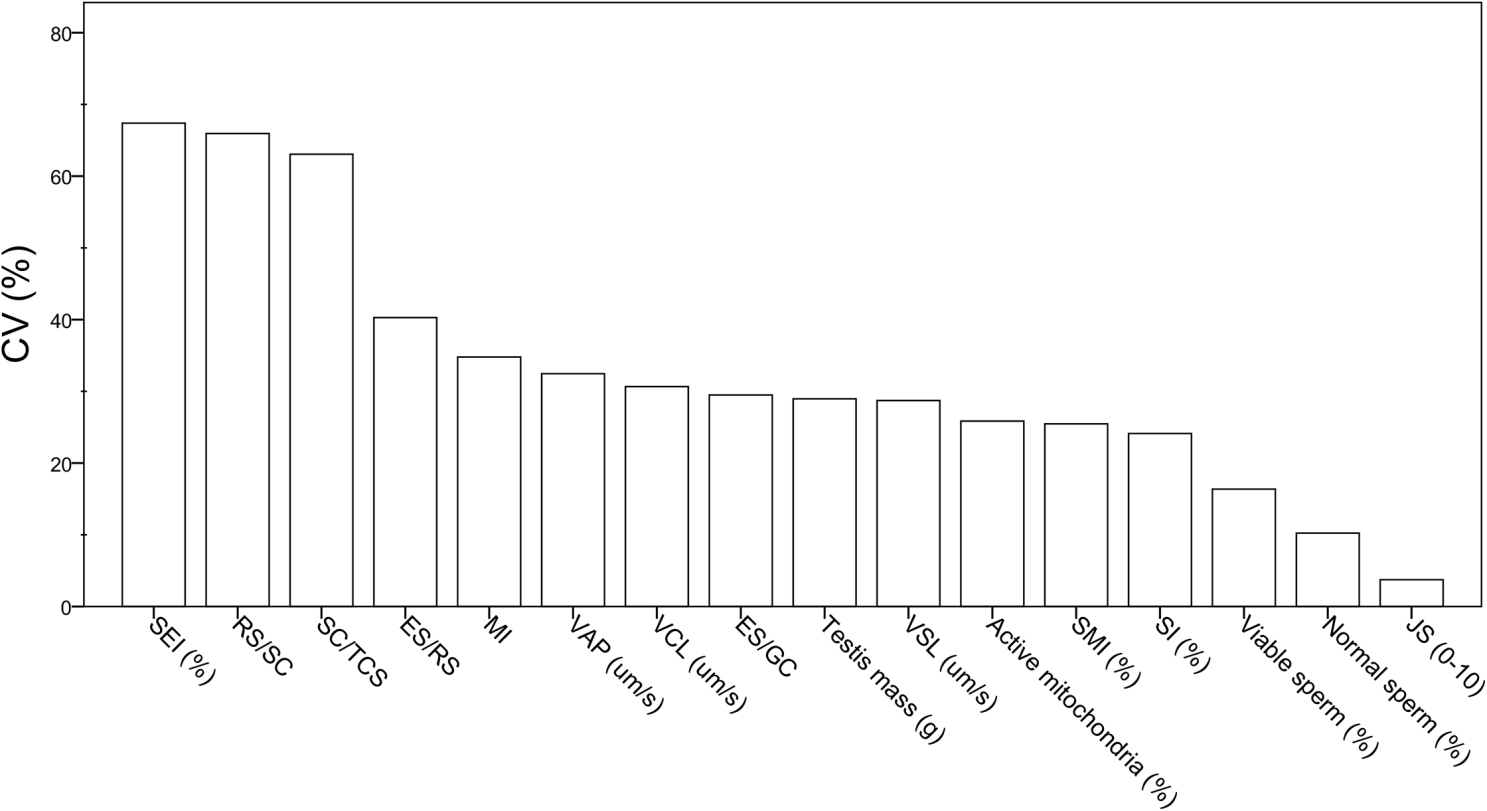
Coefficients of variation of testis mass, spermatogenic indices, and epididymal sperm parameters in red deer. SEI: Sertoli cell index; RS/SC: ratio of round spermatids to Sertoli cells; SC/TCS: Sertoli cell number per tubular cross-section; ES/RS: ratio of elongated spermatids to round spermatids; MI: meiotic index; VAP: average path velocity; VCL: curvilinear velocity; ES/GC: ratio of elongated spermatids to total germ cells; VSL: straight-line velocity; SMI: sperm motility index; SI: spermatic index; JS: Johnsen score.

Table 1 summarises the descriptive statistics of testis mass, spermatogenic indices, and sperm parameters from the 47 stags used in this study. The indices related to the Sertoli cell number and function were the testicular parameters showing the greatest variability between males, whereas the sperm production (SI) and spermatogenic activity (Johnsen score) showed the lowest variability (Fig 3). The same trend was also found for these parameters within the breeding season (S2 Table). Overall, the sperm parameters showed a coefficient of variation <35% (Fig 3). Specifically, the percentage of normal and viable spermatozoa were the parameters showing the lowest coefficient of variation between males (CV <20%; Fig 3).

**Table 1:** Descriptive statistics of testis mass, spermatogenic competence, and epididymal sperm parameters in red deer.

	Mean±SD	Min-Max	*N*
*Testicular parameters*			
Testis mass (g)	37.50±10.86	18.36–62.33	47
Johnsen score (0–10)	7.96±0.30	7.36–8.39	26
SC/TCS	4.43±2.79	1.80–10.40	15
SEI (%)	12.45±8.39	2.43–39.08	47
SI (%)	19.94±4.81	9.57–29.38	47
MI	2.38±0.83	1.22–6.61	47
ES/RS	0.52±0.21	0.17–1.19	47
ES/GC	0.20±0.06	0.08–0.38	47
RS/SC	4.66±3.07	1.03–17.54	47
*Sperm parameters*			
SMI (%)	70.21±17.88	17.50–90.00	47
VAP (μm/s)	80.49±26.13	9.88–124.43	47
VCL (μm/s)	116.31±35.67	19.80–164.12	47
VSL (μm/s)	40.70±11.69	5.77–61.52	47
Viable sperm (%)	77.72±12.73	44.51–94.79	47
Active mitochondria (%)	66.59±17.22	30.18–94.06	47
Normal sperm (%)	82.35±8.43	62.67–96.67	47

SC/TCS: Sertoli cell number per tubular cross-section; SEI: Sertoli cell index; SI: spermatic index; MI: meiotic index; ES/RS: ratio of elongated spermatids to round spermatids; ES/GC: ratio of elongated spermatids to total germ cells; RS/SC: ratio of round spermatids to Sertoli cells; SMI: sperm motility index; VAP: average path velocity; VCL: curvilinear velocity; VSL: straight-line velocity.

As expected, the testis mass, spermatogenic activity (Johnsen score), Sertoli cell number (both the SEI and the SC/TCS parameters), and sperm production (SI) were significantly higher during the breeding season than during the non-breeding season (*p* <0.05 for all; S3 Table). On the other hand, higher indices of post-meiotic germ loss and spermatogenic efficiency (i.e. low ES/RS and ES/GC ratios, respectively) were found in the BS group than in the NB group (*p* <0.05; S3 Table). All sperm parameters, except for the percentage of normal sperm, were significantly higher during the breeding season than in the PB and NB groups (*p* <0.05; S3 Table). Although the percentage of normal sperm did not show any significant difference between the breeding and non-breeding seasons, there was a tendency to be greater during the former (S3 Table).

### Correlation between testis mass and spermatogenic competence

On the whole, we found that the testis mass was significantly associated with the Sertoli cell number (SC/TCS: r = 0.619, *p* = 0.007, *N* = 15 and SEI: r = 0.248, *p* = 0.047, *N* = 47; Figure A and Figure B in S1 Fig, respectively), spermatogenic activity (Johnsen score: r = 0.344, *p* = 0.043, *N* = 26; Figure C in S1 Fig), and post-meiotic germ loss (ES/RS ratio: r = -0.250, *p* = 0.045, *N* = 47; Figure D in S1 Fig), whereas no significant relationship was found with sperm production (SI, *p*>0.05).

The Sertoli cell number per tubular cross section was also significantly associated with sperm production (SI: r = 0.600, *p* = 0.009, *N* = 15; S2 Fig), spermatogenic activity (Johnsen score: r = 0.690, *p* = 0.002, *N* = 15), and meiotic index (MI: r = 0.524, *p* = 0.023, *N* = 15). We also found that a high spermatogenic activity (Johnsen score) was significantly associated with high post-meiotic germ loss and spermatogenic efficiency (ES/RS, r = -0.359, *p* = 0.036, *N* = 26 and ES/GC, r = -0.355, *p* = 0.037, *N* = 26, respectively), and that a high spermatogenic efficiency (i.e. low ES/GC) was significantly correlated with a high sperm production (SI: r = -0.390, *p* = 0.003, *N* = 47).

### Principal component analysis of sperm quality

Principal component analysis rendered two components, which overall explained 84.227% of the total variance (Table 2). The high value of the correlation matrix (KMO measure of sampling adequacy = 0.801) and the rejection of the Bartlett sphericity test (approx. chi-square = 366.077, *df* = 21, *p<*0.001) showed the proper sampling adequacy. The first component (PC1) explained 69.862% of the total variance and accounted for sperm motility, kinetics, viability, and mitochondrial activity. The first component therefore explained sperm function. On the other hand, the second component (PC2) explained 14.365% of the total variance and only accounted for the percentage of normal sperm. Thus, the second component explained the normal sperm morphology (Table 2).

**Table 2:** Results of the principal component analysis (PCA) of epididymal sperm parameters in red deer (*N* = 47).

*Sperm parameters*	PC1	*p*	PC2	*p*
SMI	0.943	**<0.001**	0.143	0.169
VAP	0.941	**<0.001**	0.210	0.078
VCL	0.958	**<0.001**	0.139	0.176
VSL	0.912	**<0.001**	0.213	0.075
Viable sperm	0.821	**<0.001**	0.087	0.282
Active mitochondria	0.751	**<0.001**	-0.233	0.057
Normal sperm	0.110	0.230	0.965	**<0.001**
Eigenvalue	4.890		1.006	
Variance explained (%)	69.862		14.365	

SMI: sperm motility index; VAP: average path velocity; VCL: curvilinear velocity; VSL: straight-line velocity. The components were rotated (varimax) with Kaiser normalization.

### Correlations between testis mass, spermatogenic competence, and sperm quality

**Fig 4:**
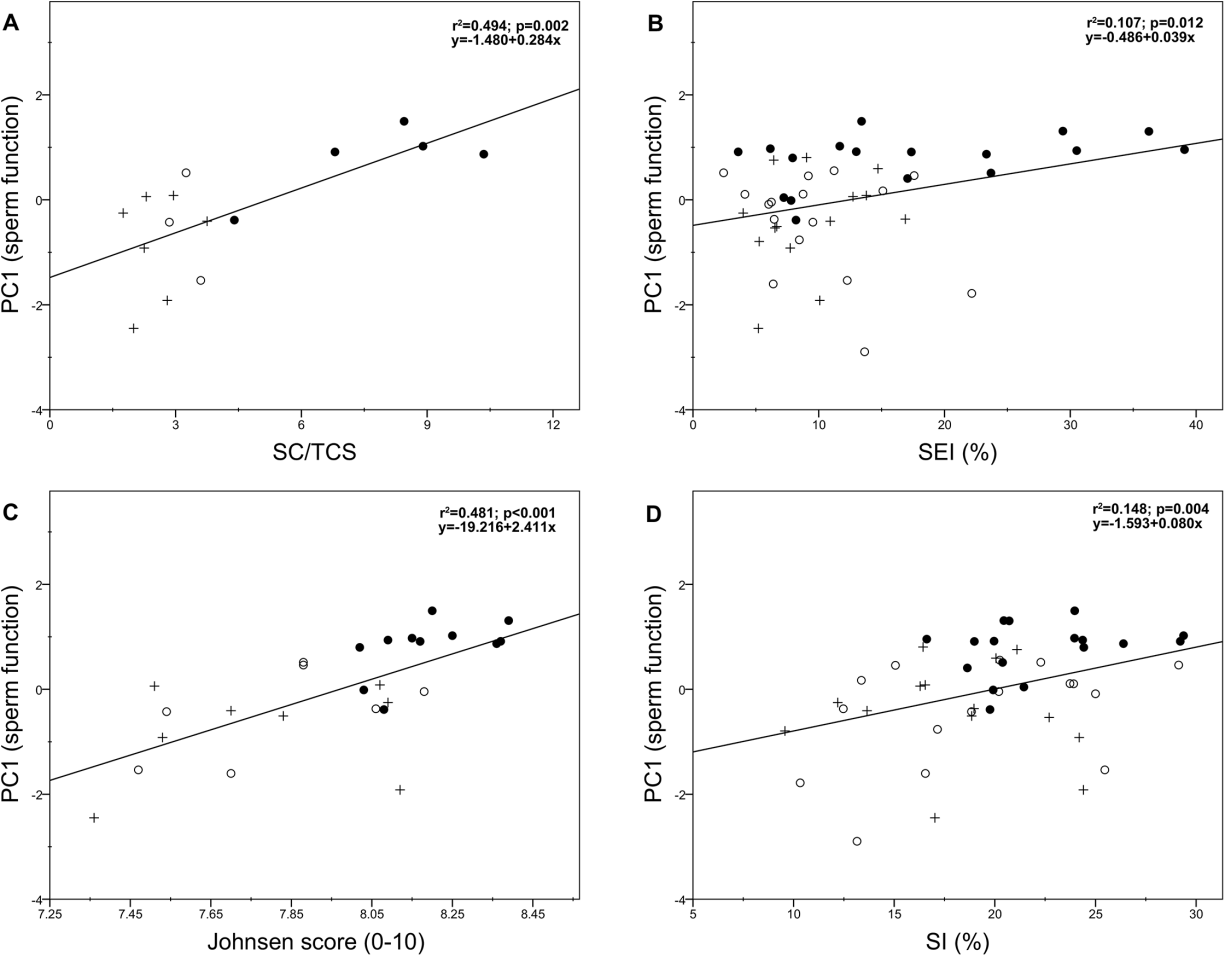
Relationships between epididymal sperm function (PC1) and Sertoli cell number (A and B), spermatogenic activity (C), and sperm production (D) in red deer culled during the breeding season (●), the post-breeding season (○), and the non-breeding season (+). SC/TCS: Sertoli cell number per tubular cross-section; SEI: Sertoli cell index; SI: spermatic index.

**Fig 5:**
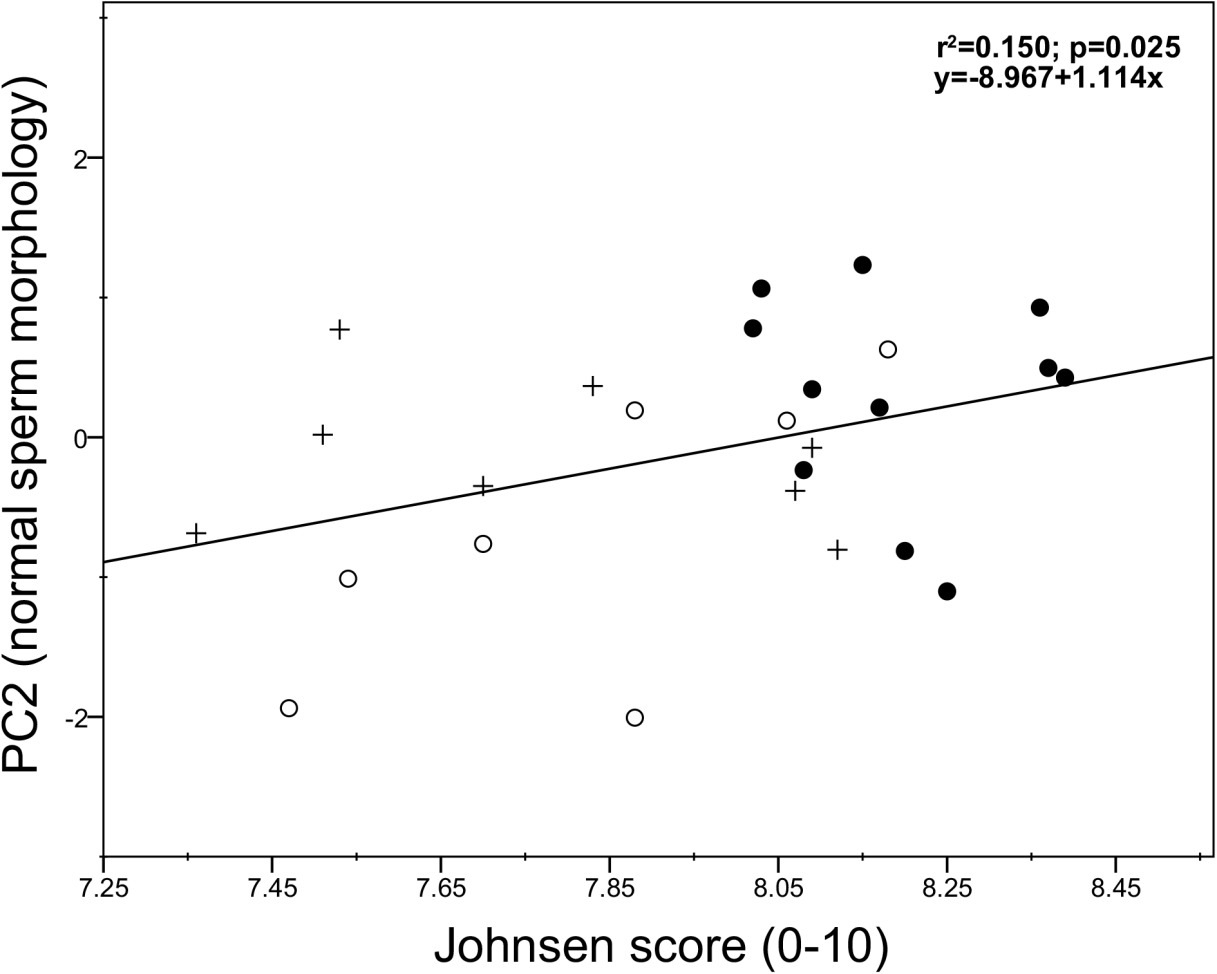
Relationship between epididymal sperm morphology (PC2) and spermatogenic activity (Johnsen score) in red deer culled during the breeding season (●), the post-breeding season (○), and the non-breeding season (+).

Overall sperm function (PC1) was positively associated with the Sertoli cell number (SC/TCS: r = 0.703, *p* = 0.002, *N* = 15 and SEI: r = 0.328, *p* = 0.012, *N* = 47; Fig 4A and 4B, respectively), spermatogenic activity (Johnsen score: r = 0.694, *p*<0.001, *N* = 26; Fig 4C), and sperm production (SI: r = 0.384, *p* = 0.004, *N* = 47; Fig 4D). In particular, the Sertoli cell number per tubular cross-section, spermatogenic activity (Johnsen score), and sperm production (SI) were positively associated with the sperm motility index (SMI), sperm kinetics (i.e. VAP and VCL), and the percentages of viable sperm and active mitochondria (all *p* <0.05; Table 3). We also found that a high sperm function was associated with a high efficiency of spermatogenesis (i.e. low ES/GC ratio) and post-meiotic germ loss (i.e. low ES/RS ratio), although the latter was not statistically significant (r = -0.309, *p* = 0.017, *N* = 47 and *r* = -0.241, *p* = 0.051, *N* = 47, respectively). Moreover, correlation analysis indeed revealed that a high efficiency of spermatogenesis (i.e. low ES/GC) and post-meiotic germ loss (i.e. low ES/RS ratio) were significantly associated with the VSL (*r* = -0.292, *p* = 0.023, *N* = 47 and *r* = -0.247, *p* = 0.047, *N* = 47, respectively; Table 3). On the other hand, testis mass did not show any significant correlation with any epididymal sperm parameter (*p*>0.05; Table 3). We finally found that normal sperm morphology (PC2) was positively correlated with spermatogenic activity (Johnsen score: r = 0.387, *p* = 0.025, *N* = 26; Fig 5), whereas no significant correlation was found with the remaining parameters (all *p*>0.05).

**Table 3:** Correlations between testis mass, spermatogenic indices, and epididymal sperm parameters in red deer (*N* = 47).

	SMI	VAP	VCL	VSL	Viable sperm	Active mitochondria	Normal sperm
Testis mass	0.17	0.15	0.11	0.17	0.03	0.07	0.15
Johnsen score ^§^	**0.66** ***	**0.73** ***	**0.68** ***	**0.65** ***	**0.46** **	**0.67** ***	**0.52** **
SC/TCS^‡^	**0.66** **	**0.74** **	**0.68** **	**0.57** *	**0.58** *	**0.64** **	0.25
SEI	0.20	**0.37** **	**0.27** *	0.24	0.20	**0.40** **	-0.07
SI	**0.33** *	**0.28** *	**0.35** **	0.22	**0.45** **	**0.36** **	-0.07
MI	0.09	0.12	0.12	0.06	0.12	0.22	-0.02
ES/RS	-0.19	-0.24	-0.24	**-0.25** *	-0.15	-0.21	-0.02
ES/GC	**-0.25** *	**-0.29** *	**-0.31** *	**-0.29** *	**-0.24** *	-0.24	-0.00
RS/SC	-0.02	-0.13	-0.06	-0.07	-0.11	-0.23	0.12

SC/TCS: Sertoli cell number per tubular cross-section; SEI: Sertoli cell index; SI: spermatic index; MI: meiotic index; ES/RS: ratio of elongated spermatids to round spermatids; ES/GC: ratio of elongated spermatids to total germ cells; RS/SC: ratio of round spermatids to Sertoli cells; SMI: sperm motility index; VAP: average path velocity; VCL: curvilinear velocity; VSL: straight-line velocity.

^§^ *N* = 26.

^‡^ *N* = 15.

Significant correlations are shown in bold

* *p*<0.05

** *p*<0.01

*** *p*<0.001.

## Discussion

To the best of our knowledge, this is the first report to show that the Sertoli cell number and spermatogenic competence covary with testis mass and sperm quality throughout different phases of gonadal activity in red deer. Our findings set the stage for the possibility that intraspecific variation in the components of spermatogenesis may contribute to the understanding of the variation in male reproductive fitness. Consistent with what was reported in other species (in bull: [39]; in stallion: [40]; in boar: [41]), our findings support that the Sertoli cell number is important in determining testis size and sperm production in red deer.

The Sertoli cell number varies between [42] and within species [41] and may explain, at least to some extent, the intra-specific variation of male reproductive traits [6].We indeed found that testis size varies considerably among red deer stags, as reported previously [22–24]. Our findings also reveal that the Sertoli cell number is positively associated with sperm parameters in red deer, supporting what was recently found by Rajak *et al*. [9] in bull. Therefore, changes in the Sertoli cell number not only affect the magnitude of sperm production [43], but also sperm function. Sertoli cells might be essential for several processes, but their clearest effect is on the elongation of spermatids and the formation of sperm tails [44]. As shown by *in vitro* studies, in the absence of Sertoli cells, spermatogenesis may progress to the early spermiogenesis stage, but round spermatids did not develop the flagellum [45]. This finding suggests that the Sertoli cells play a major role in the development of flagellum, which in turn influences sperm velocity [46], a major determinant of male fertilization success [18]. Male fertility has seldom been studied in natural populations because it has been assumed that strong selection would result in uniformly high values across males (reviewed by [47]). However, findings from natural populations of red deer have shown that stags differ markedly in their fertility and revealed a substantial degree of variation in several sperm traits [22, 47]. At least to some extent, such considerable variation may be explained by the Sertoli cell number, as the latter and fertilization success are both related with sperm kinetics in this species.

Although cell losses during spermiogenesis are considered minimal and not significant [42], we found that they significantly increase with testis size and even tend to be correlated with sperm function in red deer. High germ cell losses during the post-meiotic phase of spermatogenesis are characterised by a low ratio of elongated spermatids to round spermatids, which is associated with a high level of sperm competition in New World Blackbirds [13]. The expected ES/RS ratio is indeed theoretically 1 because spermiogenesis does not involve any further cellular division [48]. During mammalian spermatogenesis, more than half of the differentiating spermatogenic cells undergo apoptosis before they mature into spermatozoa and are phagocytosed by the Sertoli cells (reviewed by [49]). In felids, for instance, teratospermia (i.e. less than 40% of morphologically normal sperm in the ejaculate) is associated with low germ cell apoptosis and fewer Sertoli cells per tubular cross section [50, 51]. The Sertoli cell is phagocytotic and helps to remove spermatid cytoplasm through the tubulobulbar complexes and by phagocytosis of the residual bodies and degenerating germ cells [52]. It has been suggested that the phagocytic clearance of apoptotic spermatogenic cells is necessary for the progress of spermatogenesis, and thus for efficient sperm production [49], but it may also provide energy substrates to the Sertoli cells [53]. However, we have to bear in mind that the ES/GC and ES/RS indices only quantify the amount of germ cell losses without differentiating the mechanisms underlying such a phenomenon, which deserves further investigation. We have previously found that the ES/RS ratio is significantly lower during the breeding season, suggesting that highly selective spermiogenesis may reduce low-quality sperm in red deer [23]. Here, we provide evidence that a more selective spermatogenesis and spermiogenesis are significantly associated with the VSL, which is strongly associated with male fertility in red deer [18, 22].

Across the animal kingdom, male fertilisation ability is influenced by a set of sperm traits that, after controlling for sperm number, are often referred to as sperm quality [54–56]. In red deer, for instance, sperm kinetics (VAP, VCL, and VSL) and the percentage of normal sperm are positively associated with male fertility [18, 22]. In addition to these parameters, we decided to include in our analysis the sperm motility index, viability, and mitochondrial activity because of their potential implications on sperm function and fertilisation ability (e.g. [57]). Then, we performed a PCA to reduce the several sperm variables to a smaller subset that define sperm quality. Moreover, PCA reveals patterns in the data that could not be found by analyzing each variable separately [37]. Because sperm are costly to produce [58–60], theory predicts a trade-off between sperm traits occurring potentially as a consequence of either the partitioning of resources or genetic constraints (reviewed by [54]). On the other hand, sperm traits can evolve independently but in the same direction (reviewed by [61]), because they share the same goal and are the final product of an efficient spermatogenesis. In spite of some discrepancies in what sperm number and quality parameters are considered, our findings are consistent with inter- [14, 62, 63] and intra-specific [9, 22, 64] studies supporting that sperm quantity and quality are correlated with one another most likely to maximise sperm fertilisation efficiency in red deer.

Testicular functions are often based on semen analysis despite the fact that the quality of the ejaculate is the result not only of the functionality of the testicular glands, but also of the epididymal and accessory glands [65]. The effects of seminal fluids may indeed affect sperm quality and thereby the outcome of sperm competition (e.g. [66]). Moreover, epididymal sperm are functionally mature and potentially able to fertilize eggs when inseminated into the female tract [67]. In this way, the analysis of epididymal sperm allows for the elimination of any confounding effects related to accessory gland secretion. Another factor to take into account is that the duration of spermatogenesis varies considerably among species [68], but it is constant within any of them [1, 65], controlled by germ cell genotype and unaffected by natural phenomena or experimental manipulation [69]. Given that spermatogenesis in mammals usually lasts between 30 and 75 days (e.g. [65]) and that epididymal transit takes 1 or 2 weeks in most species [70], the same time lag was likely to occur between sperm production and collection in all of the samples analysed in the present study. Moreover, the biological shift from growth and differentiation of germ cells to cell death occurs during testicular regression [71], and makes this step of testicular development suitable to establish the links between spermatogenic competence and sperm output. Nevertheless, further investigations need to be extended throughout the whole reproductive cycle including hormonal assessment (e.g. FSH, inhibin B, and testosterone), which may provide further insights into the underlying mechanisms that regulate Sertoli cell function and spermatogenic efficiency.

In conclusion, we have shown here the strength of association between the competence of spermatogenesis and sperm traits in red deer. Although our results need to be considered with some caution because of the lack of the hormonal evaluation and the restricted time lapse considered, we found that not only does the Sertoli cell number affect testis size and sperm output, but also sperm function. The number of spermatozoa produced and their functionality were also positively correlated most likely to optimise male fertilisation ability in red deer. Our findings contribute to the understanding of the dynamics of spermatogenesis in a seasonal breeding mammal, and reveal new insights into the role of testicular function and Sertoli cell number on sperm quantity and quality.

## Supporting Information

S1 Fig Relationships between testis mass and Sertoli cell number (Figures A and B), spermatogenic activity (Figure C), and post-meiotic germ loss (Figure D) in red deer culled during the breeding season (●), the post-breeding season (○), and the non-breeding season (+). SC/TCS: Sertoli cell number per tubular cross-section; SEI: Sertoli cell index; ES/RS: ratio of elongated spermatids to round spermatids.
(EPS)

S2 Fig Relationship between the Sertoli cell number per tubular cross-section and sperm production in red deer culled during the breeding season (●), the post-breeding season (○), and the non-breeding season (+). SI: spermatic index; SC/TCS: Sertoli cell number per tubular cross-section.
(EPS)

S1 File Dataset. Testicular and sperm parameters in red deer.
(XLS)

S1 Table Comparison of testicular parameters between the right and left testis. SC/TCS: Sertoli cell number per tubular cross-section; SEI: Sertoli cell index; SI: spermatic index; MI: meiotic index; ES/RS: ratio of elongated spermatids to round spermatids; ES/GC: ratio of elongated spermatids to total germ cells; RS/SC: ratio of round spermatids to Sertoli cells. Data are shown as the mean±SD.
(DOCX)

S2 Table Coefficients of variation of testis mass, spermatogenic competence, and epididymal sperm parameters in red deer throughout different reproductive phases. BS: Breeding season; PB: Post-Breeding season; NB: Non-Breeding season; SC/TCS: Sertoli cell number per tubular cross-section; SEI: Sertoli cell index; SI: spermatic index; MI: meiotic index; ES/RS: ratio of elongated spermatids to round spermatids; ES/GC: ratio of elongated spermatids to total germ cells; RS/SC: ratio of round spermatids to Sertoli cells; SMI: sperm motility index; VAP: average path velocity; VCL: curvilinear velocity; VSL: straight-line velocity. ^§^ *N* = 26: in the BS group *n* = 11, in the PB group *n* = 7, and in the NB group *n* = 8, respectively. ^‡^ *N* = 15: in the BS group *n* = 5, in the PB group *n* = 3, and in the NB group *n* = 7, respectively. Coefficients of variation are shown as a percentage (%).
(DOCX)

S3 Table Testis mass, spermatogenic indices, and epididymal sperm parameters in red deer across different reproductive phases. BS: Breeding season; PB: Post-Breeding season; NB: Non-Breeding season; SC/TCS: Sertoli cell number per tubular cross-section; SEI: Sertoli cell index; SI: spermatic index; MI: meiotic index; ES/RS: ratio of elongated spermatids to round spermatids; ES/GC: ratio of elongated spermatids to total germ cells; RS/SC: ratio of round spermatids to Sertoli cells; SMI: sperm motility index; VAP: average path velocity; VCL: curvilinear velocity; VSL: straight-line velocity. ^§^ *N* = 26: in the BS group *n* = 11, in the PB group *n* = 7, and in the NB group *n* = 8, respectively. ^‡^ *N* = 15: in the BS group *n* = 5, in the PB group *n* = 3, and in the NB group *n* = 7, respectively. Data are shown as the mean±SD. Different superscripts within the same row are statistically different (*p*<0.05).
(DOCX)

## References

[1] Hess RA, Franca LR. Spermatogenesis and cycle of the seminiferous epithelium In: Cheng CY, editor. Molecular mechanisms in spermatogenesis. New York: Springer; 2008 pp. 1–15. 10.1007/978-0-387-09597-4_1 19856159

[2] De Kretser DM, Loveland KL, Meinhardt A, Simorangkir D, Wreford N. Spermatogenesis. Hum Reprod. 1998; 1: 1–8. (10.1093/humrep/13.suppl_1.1) 9663765

[3] Pudney J. Spermatogenesis in nonmammalian vertebrates. Microsc Res Tech. 1995; 32: 459–497. (10.1002/jemt.1070320602) 8605396

[4] Dadhich RK, Barrionuevo FJ, Real FM, Lupiañez DG, Ortega E, Burgos M, et al Identification of live germ-cell desquamation as a major mechanism of seasonal testis regression in mammals: a study in the Iberian mole (*Talpa occidentalis*). Biol Reprod. 2013; 88: 1–12. 23515671 10.1095/biolreprod.112.106708

[5] Sharpe RM. Regulation of spermatogenesis In: Knobil E, Neill JD, editors. The physiology of reproduction. San Diego: Elsevier Academic Press; 1994 pp. 1363–1434.

[6] Sharpe RM, McKinnell C, Kivlin C, Fisher JS. Proliferation and functional maturation of Sertoli cells, and their relevance to disorders of testis function in adulthood. Reproduction. 2003; 125: 769–784. (10.1530/rep.0.1250769) 12773099

[7] Johnson L, Thompson DLJr, Varner DD. Role of Sertoli cell number and function on regulation of spermatogenesis. Anim Reprod Sci. 2008; 105: 23–51. (10.1016/J.ANIREPROSCI.2007.11.029) 18242891

[8] Mossman JA, Pearson JT, Moore HD, Pacey AA. Variation in mean human sperm length is linked with semen characteristics. Hum Reprod. 2013; 28: 22–32. (10.1093/humrep/des382) 23108349

[9] Rajak SK, Kumaresan A, Gaurav MK, Layek SS, Mohanty TK, Aslam MKM, et al Testicular cell indices and peripheral blood testosterone concentrations in relation to age and semen quality in crossbred (Holstein Friesian×Tharparkar) bulls. Asian-Australas J Anim Sci. 2014; 27: 1554–1561. (10.5713/ajas.2014.14139) 25358314 PMC4213699

[10] Klonisch T, Schön J, Hombach-Klonisch S, Blottner S. The roe deer as a model for studying seasonal regulation of testis function. Int J Androl. 2006; 29: 122–128. (10.1111/J.1365-2605.2005.00603.X) 16371113

[11] Schärer L, Da Lage JL, Joly D. Evolution of testicular architecture in the Drosophilidae: a role for sperm length. BMC Evol Biol. 2008; 8: 143 (10.1186/1471-2148-8-143) 18477397 PMC2396631

[12] Lüpold S, Linz GM, Rivers JW, Westneat DF, Birkhead TR. Sperm competition selects beyond relative testes size in birds. Evolution. 2009; 63: 391–402. (10.1111/j.1558-5646.2008.00571.x.) 19215291

[13] Lüpold S, Wistuba J, Damm OS, Rivers JW, Birkhead TR. Sperm competition leads to functional adaptations in avian testes to maximize sperm quantity and quality. Reproduction. 2011; 141: 595–605. (10.1530/REP-10-0501) 21307271

[14] Rowe M, Pruett-Jones S. Sperm competition selects for sperm quantity and quality in the Australian Maluridae. PLoS One. 2011; 6(1):e15720 (10.1371/journal.pone.0015720) 21283577 PMC3026798

[15] Goeritz F, Quest M, Wagener A, Fassbender M, Broich A, Hildebrandt TB, et al Seasonal timing of sperm production in roe deer: interrelationship among changes in ejaculate parameters, morphology and functions of testis and accessory glands. Theriogenology. 2003; 59: 1487–1502. (10.1016/S0093-691X(02)01201-3) 12559454

[16] Schulte-Hostedde AI, Millar JS. Intraspecific variation of testis size and sperm length in the yellow-pine chipmunk (*Tamias amoenus*): implications for sperm competition and reproductive success. Behav Ecol Sociobiol. 2004; 55: 272–277. (10.1007/s00265-003-0707-z)

[17] Gomendio M, Roldan ER. Coevolution between male ejaculates and female reproductive biology in eutherian mammals. Proc R Soc B. 1993; 252: 7–12. (10.1098/rspb.1993.0039) 8389048

[18] Gomendio M, Roldan ER. Implications of diversity in sperm size and function for sperm competition and fertility. Int J Dev Biol. 2008; 52: 439–447. (10.1387/ijdb.082595mg) 18649256

[19] Clutton-Brock TH, Guinness FE, Albon SD. Red deer: Behaviour and ecology of two sexes 1 st ed. Chicago: Chicago University Press; 1982.

[20] Malo AF, Roldan ER, Garde J, Soler AJ, Gomendio M. Antlers honestly advertise sperm production and quality. Proc R Soc B. 2005; 272: 149–157. (10.1098/rspb.2004.2933) 15695205 PMC1634960

[21] Guinness F, Lincoln GA, Short RV. Reproductive cycle of female red deer, *Cervus elaphus* L. J Reprod Fertil. 1971; 27: 427–438. 5167355 10.1530/jrf.0.0270427

[22] Malo AF, Garde JJ, Soler AJ, García AJ, Gomendio M, Roldan ER. Male fertility in natural populations of red deer is determined by sperm velocity and the proportion of normal spermatozoa. Biol Reprod. 2005; 72: 822–829. (10.1095/biolreprod.104.036368) 15576823

[23] Pintus E, Ros-Santaella JL, Garde JJ. Variation of spermatogenic and Sertoli cell number detected by fine needle aspiration cytology (FNAC) in Iberian red deer during and out of breeding season. Reprod Fertil Dev. 2015; 27: 812–822. (10.1071/RD13419) 25483787

[24] Martinez-Pastor F, Guerra C, Kaabi M, Garcia-Macias V, de Paz P, Alvarez M, et al Season effect on genitalia and epididymal sperm from Iberian red deer, roe deer and Cantabrian chamois. Theriogenology. 2005; 63: 1857–1875. (10.1016/j.theriogenology.2004.08.006) 15823344

[25] Ramm SA, Schärer L. The evolutionary ecology of testicular function: size isn't everything. Biol Rev. 2014; 89: 874–888. (10.1111/brv.12084) 24495304

[26] Fernández-Santos MR, Esteso MC, Montoro V, Soler AJ, Garde JJ. Cryopreservation of Iberian red deer (*Cervus elaphus hispanicus*) epididymal spermatozoa: effects of egg yolk, glycerol and cooling rate. Theriogenology. 2006; 66: 1931–1942. (10.1016/j.theriogenology.2006.05.012) 16759687

[27] Domínguez-Rebolledo AE, Fernández-Santos MR, Bisbal A, Ros-Santaella JL, Ramón M, Carmona M, et al Improving the effect of incubation and oxidative stress on thawed spermatozoa from red deer by using different antioxidant treatments. Reprod Fertil Dev. 2010; 22: 856–870. (10.1071/RD09197) 20450838

[28] Soler AJ, Garde JJ. Relationship between the characteristics of epididymal red deer spermatozoa and penetrability into zona-free hamster ova. J Androl. 2003; 24: 393–400. (10.1002/j.1939-4640.2003.tb02688.x) 12721216

[29] Vahed K, Parker DJ. The evolution of large testes: sperm competition or male mating rate? Ethology. 2012; 118: 107–117. (10.1111/j.1439-0310.2011.01991.x)

[30] Leme DP, Papa FO. Cytological identification and quantification of testicular cell types using fine needle aspiration in horses. Equine Vet J. 2000; 32: 444–446 (10.2746/042516400777591156) 11037268

[31] Segatelli TM, Franca LR, Pinheiro PFF, Almeida CCD, Martinez M, Martinez FE. Spermatogenic cycle length and spermatogenic efficiency in the gerbil (*Meriones unguiculatus*). J Androl. 2004; 25: 872–880. (10.1002/j.1939-4640.2004.tb03156.x) 15477358

[32] Latendresse JR, Warbrittion AR, Jonassen H & Creasy DM 2002 Fixation of testes and eyes using a modified Davidson’s fluid: comparison with Bouin’s fluid and conventional Davidson’s fluid. Toxicol Pathol 30 524–533. (10.1080/01926230290105721) 12187944

[33] Howroyd P, Hoyle-Thacker R, Light O, Williams D, Kleymenova E. Morphology of the fetal rat testis preserved in different fixatives. Toxicol Pathol. 2005; 33: 300–304. (10.1080/01926230590896145) 15902974

[34] Johnsen SG. Testicular biopsy score count-a method for registration of spermatogenesis in human testes: normal values and results in 335 hypogonadal males. Hormones. 1970; 1: 2–25. (10.1159/000178170) 5527187

[35] Garde JJ, Ortiz N, Garcia AJ, Gallego L, Landete-Castillejos T, Lopez A. Postmortem assessment of sperm characteristics of the red deer during the breeding season. Arch Androl. 1998; 41: 195–202. (10.3109/01485019808994891) 9805148

[36] Comizzoli P, Mauget R, Mermillod P. Assessment of in vitro fertility of deer spermatozoa by heterologous IVF with zona-free bovine oocytes. Theriogenology. 2001; 56: 261–274. (10.1016/S0093-691X(01)00561-1) 11480618

[37] Quinn G and Keough M. 2002 Experimental design and data analysis for biologists Cambridge University Press; 2002 pp. 443–472.

[38] Budaev SV. Using Principal Components and Factor Analysis in Animal Behaviour Research: Caveats and Guidelines. Ethology. 2010; 116: 472–480. (10.1111/j.1439-0310.2010.01758.x)

[39] Berndtson WE, Igboeli G, Parker WG. The numbers of Sertoli cells in mature Holstein bulls and their relationship to quantitative aspects of spermatogenesis. Biol Reprod. 1987; 37: 60–67. (10.1095/biolreprod37.1.60) 3651551

[40] Johnson L, Carter GK, Varner DD, Taylor TS, Blanchard TL, Rembert MS. The relationship of daily sperm production with number of Sertoli cells and testicular size in adult horses: role of primitive spermatogonia. J Reprod Fertil. 1994; 100: 315–321. (10.1530/jrf.0.1000315) 8182606

[41] Okwun OE, Igboeli G, Ford JJ, Lunstra DD, Johnson L. Number and function of Sertoli cells, number and yield of spermatogonia, and daily sperm production in three breeds of boar. J Reprod Fertil. 1996; 107: 137–149. (10.1530/jrf.0.1070137) 8699427

[42] Johnson L, Varner DD, Roberts ME, Smith TL, Keillor GE, Scrutchfield WL. Efficiency of spermatogenesis: a comparative approach. Anim Reprod Sci. 2000; 60: 471–480. (10.1016/S0378-4320(00)00108-1) 10844217

[43] Holsberger DR, Cooke PS. Understanding the role of thyroid hormone in Sertoli cell development: a mechanistic hypothesis. Cell Tissue Res. 2005; 322: 133–140. (10.1007/s00441-005-1082-z) 15856309

[44] Griswold MD. Perspective on the function of Sertoli cells In: Skinner MK, Griswold MD, editors. Sertoli cell biology. San Diego: Elsevier Academic Press; 2005 pp. 15–18.

[45] Feng LX, Chen Y, Dettin L, Pera RA, Herr JC, Goldberg E, et al Generation and in vitro differentiation of a spermatogonial cell line. Science. 2002; 297: 392–395. (10.1126/science.1073162) 12077424

[46] Malo AF, Gomendio M, Garde J, Lang-Lenton B, Soler AJ, Roldan ER. Sperm design and sperm function. Biol Lett. 2006; 2: 246–249. (10.1098/rsbl.2006.0449) 17148374 PMC1618917

[47] Gomendio M, Malo AF, Garde J, Roldan ER. Sperm traits and male fertility in natural populations. Reproduction. 2007; 134: 19–29. (10.1530/REP-07-0143) 17641085

[48] Luetjens CM, Weinbauer GF, Wistuba J. Primate spermatogenesis: new insights into comparative testicular organisation, spermatogenic efficiency and endocrine control. Biol Rev Camb Philos Soc. 2005; 80: 475–488. (10.1017/S1464793105006755) 16094809

[49] Nakanishi Y, Shiratsuchi A. Phagocytic removal of apoptotic spermatogenic cells by Sertoli cells: mechanisms and consequences. Biol Pharm Bull. 2004; 27: 13–16. (10.1248/bpb.27.13) 14709891

[50] Neubauer K, Jewgenow K, Blottner S, Wildt DE, Pukazhenthi BS. Quantity rather than quality in teratospermic males: a histomorphometric and flow cytometric evaluation of spermatogenesis in the domestic cat (Felis catus). Biol Reprod. 2004; 71: 1517–1524. (10.1095/biolreprod.104.031062) 15229134

[51] Jewgenow K, Neubauer K, Blottner S, Schön J, Wildt DE, Pukazhenthi BS. Reduced germ cell apoptosis during spermatogenesis in the teratospermic domestic cat. J Androl. 2009; 30: 460–468. (10.2164/jandrol.108.006726) 19201698

[52] Hess RA, França LR. Structure of the Sertoli cell In: Skinner MK, Griswold MD, editors. Sertoli cell biology. San Diego: Elsevier Academic Press; 2005 pp. 19–40.

[53] Xiong W, Wang H, Wu H, Chen Y, Han D. Apoptotic spermatogenic cells can be energy sources for Sertoli cells. Reproduction. 2009; 137: 469–479. (10.1530/REP-08-0343) 19074501

[54] Snook RR. Sperm in competition: not playing by the numbers. Trends Ecol Evol. 2005; 20: 46–53. (10.1016/j.tree.2004.10.011) 16701340

[55] Pizzari T, Parker GA. Sperm competition and sperm phenotype In: Birkhead TR, Hosken DJ, Pitnick S, editors. Sperm Biology: An Evolutionary Perspective. Oxford: Academic Press; 2009 pp. 207–245.

[56] Fitzpatrick JL, Lüpold S. Sexual selection and the evolution of sperm quality. Mol Hum Reprod. 2014; 20: 1180–1189. (10.1093/molehr/gau067) 25323970

[57] Amaral A, Lourenço B, Marques M, Ramalho-Santos J. Mitochondria functionality and sperm quality. Reproduction. 2013; 146: 163–174. (10.1530/REP-13-0178) 23901129

[58] Dewsbury DA. Ejaculate cost and male choice. Am Nat. 1982; 119: 601–610. (10.1086/283938)

[59] Wedell N, Gage MJG, Parker GA. Sperm competition, male prudence and sperm-limited females. Trends Ecol & Evol. 2002; 17: 313–320.

[60] Thomsen R, Soltis J, Matsubara M, Matsubayashi K, Onuma M, Takenaka O. How costly are ejaculates for Japanese macaques? Primates. 2006; 47: 272–274. (10.1007/s10329-005-0171-7) 16467956

[61] Parker GA, Pizzari T. Sperm competition and ejaculate economics. Biol Rev Camb Philos Soc 2010; 85: 897–934. (10.1111/j.1469-185X.2010.00140.x) 20560928

[62] Gómez Montoto L, Magaña C, Tourmente M, Martín-Coello J, Crespo C, Luque-Larena JJ, et al Sperm competition, sperm numbers and sperm quality in muroid rodents. PLoS One. 2011; 6(3):e18173 (10.1371/journal.pone.0018173) 21464956 PMC3064651

[63] Lüpold S. Ejaculate quality and constraints in relation to sperm competition levels among eutherian mammals. Evolution. 2013; 67: 3052–3060. (10.1111/evo.12132) 24094354

[64] Firman RC, Simmons LW. Experimental evolution of sperm quality via postcopulatory sexual selection in house mice. Evolution. 2010; 64: 1245–1256. (10.1111/j.1558-5646.2009.00894.x) 19922447

[65] Amann RP. A critical review of methods for evaluation of spermatogenesis from seminal characteristics. J Androl. 1981; 2: 37–58. (10.1002/j.1939-4640.1981.tb00595.x)

[66] Cameron E, Day T, Rowe L. Sperm competition and the evolution of ejaculate composition. Am Nat. 2007; 169: E158–E172. (10.1086/516718) 17479456

[67] Cooper TG. Sperm maturation in the epididymis: a new look at an old problem. Asian J Androl. 2007; 9: 533–539. (10.1111/j.1745-7262.2007.00285.x) 17589792

[68] Peirce EJ, Breed WG. A comparative study of sperm production in two species of Australian arid zone rodents (*Pseudomys australis*, *Notomys alexis*) with marked differences in testis size. Reproduction. 2001; 121: 239–247. (10.1530/rep.0.1210239) 11226048

[69] França LR, Ogawa T, Avarbock MR, Brinster RL, Russell LD. Germ cell genotype controls cell cycle during spermatogenesis in the rat. Biol Reprod. 1998; 59: 1371–1377. (10.1095/biolreprod59.6.1371) 9828180

[70] Cooper TG. The epididymis, cytoplasmic droplets and male fertility. Asian J Androl. 2011; 13: 130–138. (10.1038/aja.2010.97) 21076437 PMC3739406

[71] Griswold MD, McLean D. The Sertoli cell. In: Knobil E, Neill JD, editors. The physiology of reproduction. San Diego: Elsevier Academic Press; 1994 pp. 949–975.

