# Supplementary material for: Beyond Testis Size: Links between Spermatogenesis and Sperm Traits in a Seasonal Breeding Mammal
Source: PLoS One. 2015 Oct 2;10(10):e0139240. doi: 10.1371/journal.pone.0139240 (PMC4592251; doi:10.1371/journal.pone.0139240)
Supplement: S1 Table — SC/TCS: Sertoli cell number per tubular cross-section; SEI: Sertoli cell index; SI: spermatic index; MI: meiotic index; ES/RS: ratio of elongated spermatids to round spermatids; ES/GC: ratio of elongated spermatids to total germ cells; RS/SC: ratio of round spermatids to Sertoli cells. Data are shown as the mean±SD. (DOCX) [file pone.0139240.s004.docx]

**S1 Table. Comparison of testicular parameters between the right and left testis.**

|  | **Right testis** | **Left testis** | ***N*** | ***p*** |
| --- | --- | --- | --- | --- |
| *Testicular parameters* |  |  |  |  |
|  |  |  |  |  |
| Testis mass (g) | 37.55±11.44 | 37.46±10.53 | 47 | 0.855 |
| Johnsen score (0-10) | 7.97±0.34 | 7.96±0.29 | 26 | 0.836 |
| SC/TCS | 4.47±3.24 | 4.38±2.61 | 15 | 0.849 |
| SEI (%) | 12.56±9.52 | 12.46±8.25 | 47 | 0.743 |
| SI (%) | 19.02±6.39 | 20.83±6.18 | 47 | 0.133 |
| MI | 2.42±1.16 | 2.45±0.94 | 47 | 0.832 |
| ES/RS | 0.53±0.23 | 0.52±0.26 | 47 | 0.874 |
| ES/GC | 0.20±0.06 | 0.20±0.07 | 47 | 0.479 |
| RS/SC | 4.97±3.53 | 4.76±3.38 | 47 | 0.216 |
|  |  |  |  |  |

SC/TCS: Sertoli cell number per tubular cross-section; SEI: Sertoli cell index; SI: spermatic index; MI: meiotic index; ES/RS: ratio of elongated spermatids to round spermatids; ES/GC: ratio of elongated spermatids to total germ cells; RS/SC: ratio of round spermatids to Sertoli cells. Data are shown as the mean±SD.
