# Supplementary material for: Beyond Testis Size: Links between Spermatogenesis and Sperm Traits in a Seasonal Breeding Mammal
Source: PLoS One. 2015 Oct 2;10(10):e0139240. doi: 10.1371/journal.pone.0139240 (PMC4592251; doi:10.1371/journal.pone.0139240)
Supplement: S2 Table — BS: Breeding season; PB: Post-Breeding season; NB: Non-Breeding season; SC/TCS: Sertoli cell number per tubular cross-section; SEI: Sertoli cell index; SI: spermatic index; MI: meiotic index; ES/RS: ratio of elongated spermatids to round spermatids; ES/GC: ratio of elongated spermatids to total germ cells; RS/SC: ratio of round spermatids to Sertoli cells; SMI: sperm motility index; VAP: average path velocity; VCL: curvilinear velocity; VSL: straight-line velocity. § N = 26: in the BS group n = 11, in the PB group n = 7, and in the NB group n = 8, respectively. ‡ N = 15: in the BS group n = 5, in the PB group n = 3, and in the NB group n = 7, respectively. Coefficients of variation are shown as a percentage (%). (DOCX) [file pone.0139240.s005.docx]

**S2 Table. Coefficients of variation of testis mass, spermatogenic competence, and epididymal sperm parameters in red deer throughout different reproductive phases.**

|  | **BS**  (*n*=17) | **PB**  (*n*=16) | **NB**  (*n*=14) |
| --- | --- | --- | --- |
| *Testicular parameters* |  |  |  |
|  |  |  |  |
| Testis mass | 32.50 | 15.71 | 21.49 |
| Johnsen score^§^ | 1.65 | 3.34 | 3.82 |
| SC/TCS^‡^ | 29.23 | 11.61 | 26.66 |
| SEI | 63.83 | 52.02 | 43.16 |
| SI | 16.41 | 28.53 | 24.32 |
| MI | 42.79 | 20.45 | 28.83 |
| ES/RS | 40.70 | 27.11 | 40.36 |
| ES/GC | 30.59 | 19.86 | 29.17 |
| RS/SC | 62.91 | 70.98 | 51.95 |
| *Sperm parameters* |  |  |  |
| SMI | 10.07 | 30.44 | 28.78 |
| VAP | 12.82 | 34.85 | 33.63 |
| VCL | 12.34 | 37.08 | 32.28 |
| VSL | 12.98 | 32.74 | 33.36 |
| Viable sperm | 9.72 | 21.02 | 12.71 |
| Active mitochondria | 21.00 | 22.19 | 25.28 |
| Normal sperm | 9.50 | 10.98 | 9.45 |

BS: Breeding season; PB: Post-Breeding season; NB: Non-Breeding season; SC/TCS: Sertoli cell number per tubular cross-section; SEI: Sertoli cell index; SI: spermatic index; MI: meiotic index; ES/RS: ratio of elongated spermatids to round spermatids; ES/GC: ratio of elongated spermatids to total germ cells; RS/SC: ratio of round spermatids to Sertoli cells; SMI: sperm motility index; VAP: average path velocity; VCL: curvilinear velocity; VSL: straight-line velocity. ^§^*N*=26: in the BS group *n*=11, in the PB group *n*=7, and in the NB group *n*=8, respectively. ^‡^*N*=15: in the BS group *n*=5, in the PB group *n*=3, and in the NB group *n*=7, respectively. Coefficients of variation are shown as a percentage (%).
