# Supplementary material for: Beyond Testis Size: Links between Spermatogenesis and Sperm Traits in a Seasonal Breeding Mammal
Source: PLoS One. 2015 Oct 2;10(10):e0139240. doi: 10.1371/journal.pone.0139240 (PMC4592251; doi:10.1371/journal.pone.0139240)
Supplement: S3 Table — BS: Breeding season; PB: Post-Breeding season; NB: Non-Breeding season; SC/TCS: Sertoli cell number per tubular cross-section; SEI: Sertoli cell index; SI: spermatic index; MI: meiotic index; ES/RS: ratio of elongated spermatids to round spermatids; ES/GC: ratio of elongated spermatids to total germ cells; RS/SC: ratio of round spermatids to Sertoli cells; SMI: sperm motility index; VAP: average path velocity; VCL: curvilinear velocity; VSL: straight-line velocity. § N = 26: in the BS group n = 11, in the PB group n = 7, and in the NB group n = 8, respectively. ‡ N = 15: in the BS group n = 5, in the PB group n = 3, and in the NB group n = 7, respectively. Data are shown as the mean±SD. Different superscripts within the same row are statistically different (p<0.05). (DOCX) [file pone.0139240.s006.docx]

**S3 Table. Testis mass, spermatogenic indices, and epididymal sperm parameters in red deer across different reproductive phases.**

|  | **BS**  (*n*=17) | **PB**  (*n*=16) | **NB**  (*n*=14) |
| --- | --- | --- | --- |
| *Testicular parameters* |  |  |  |
|  |  |  |  |
| Testis mass (g) | 41.77±13.57^a^ | 40.17±6.31^a^ | 29.28±6.29^b^ |
| Johnsen score (0-10)^§^ | 8.19±0.14^a^ | 7.82±0.26^b^ | 7.78±0.30^b^ |
| SC/TCS^‡^ | 7.78±2.27^a^ | 3.23±0.38^b^ | 2.54±0.68^b^ |
| SEI (%) | 17.39±11.10^a^ | 9.97±5.19^ab^ | 9.28±4.01^b^ |
| SI (%) | 22.27±3.65^a^ | 19.18±5.47^ab^ | 18.00±4.38^b^ |
| MI | 2.67±1.14^a^ | 2.24±0.46^a^ | 2.18±0.63^a^ |
| ES/RS | 0.43±0.18^a^ | 0.50±0.13^ab^ | 0.64±0.26^b^ |
| ES/GC | 0.17±0.05^a^ | 0.20±0.04^ab^ | 0.23±0.07^b^ |
| RS/SC | 3.54±2.23^a^ | 5.53±3.92^a^ | 5.01±2.60^a^ |
| *Sperm parameters* |  |  |  |
| SMI (%) | 81.76±8.23^a^ | 64.84±19.74^b^ | 62.32±17.93^b^ |
| VAP (µm/s) | 103.02±13.21^a^ | 67.12±23.39^b^ | 68.41±23.00^b^ |
| VCL (µm/s) | 142.18±17.54^a^ | 104.05±38.58^b^ | 98.92±31.93^b^ |
| VSL (µm/s) | 48.48±6.29^a^ | 35.63±11.67^b^ | 37.06±12.36^b^ |
| Viable sperm (%) | 85.65±8.33^a^ | 72.94±15.33^b^ | 73.55±9.35^b^ |
| Active mitochondria (%) | 78.30±16.44^a^ | 59.49±13.20^b^ | 60.47±15.29^b^ |
| Normal sperm (%) | 85.22±8.10^a^ | 82.00±9.00^a^ | 79.29±7.49^a^ |

BS: Breeding season; PB: Post-Breeding season; NB: Non-Breeding season; SC/TCS: Sertoli cell number per tubular cross-section; SEI: Sertoli cell index; SI: spermatic index; MI: meiotic index; ES/RS: ratio of elongated spermatids to round spermatids; ES/GC: ratio of elongated spermatids to total germ cells; RS/SC: ratio of round spermatids to Sertoli cells; SMI: sperm motility index; VAP: average path velocity; VCL: curvilinear velocity; VSL: straight-line velocity. ^§^*N*=26: in the BS group *n*=11, in the PB group *n*=7, and in the NB group *n*=8, respectively. ^‡^*N*=15: in the BS group *n*=5, in the PB group *n*=3, and in the NB group *n*=7, respectively. Data are shown as the mean±SD. Different superscripts within the same row are statistically different (*p*<0.05).
